# Nonfunctional Supernumerary Testis in a 26-Year-Old Man with Orchalgia

**DOI:** 10.1100/tsw.2006.384

**Published:** 2006-08-11

**Authors:** Christopher M. Simons, Moshe Wald

**Affiliations:** Department of Urology, University of Iowa, Iowa City, IA, USA

**Keywords:** orchitis, testis, genitalia, male, neoplasms, germ cell

## Abstract

Polyorchidism is a rare phenomenon, with 47 histologically confirmed cases and 77 total reported cases. In most patients, it is an incidental finding or an asymptomatic scrotal mass. More than half of histologically confirmed supernumerary testes have been reported to have the potential for sperm production. We report a case of a man with symptomatic left scrotal pain who was found to have a nonfunctional supernumerary testis in his left hemiscrotum.

## INTRODUCTION

The presence of a supernumerary testis is a rare phenomenon, with 77 cases documented in the literature to date[1]. They are often asymptomatic, but may have an association with infertility, hydrocele, inguinal hernia, testicular torsion, cryptorchidism, or malignancy[2,3]. An association with malignancy is rare, with only 5 reported cases to date[4]. Up to 65% of reported supernumerary testes have been functional in that they have had the potential to maintain spermatogenesis[1]. Our case demonstrates the more unique features of polyorchidism — germ cell aplasia and an association with scrotal pain.

## CASE REPORT AND MANAGEMENT

A 26-year-old man presented with a 6-week history of left-sided orchalgia with a palpable mass in the left hemiscrotum. He reported pain similar to that he had previously experienced when passing renal stones, except that it was persistent. He had not experienced any trauma preceding the onset of these symptoms. He had not noticed a progressive enlargement of the mass since he first detected it. He had previously been evaluated in an emergency room for the same concern. A scrotal ultrasound at that time suggested findings possibly consistent with transient left testicular torsion, and also noted a scrotal mass in the left hemiscrotum, aside from the left testis (Fig. 1). The mass was noted to have the same echogenicity as the testis.

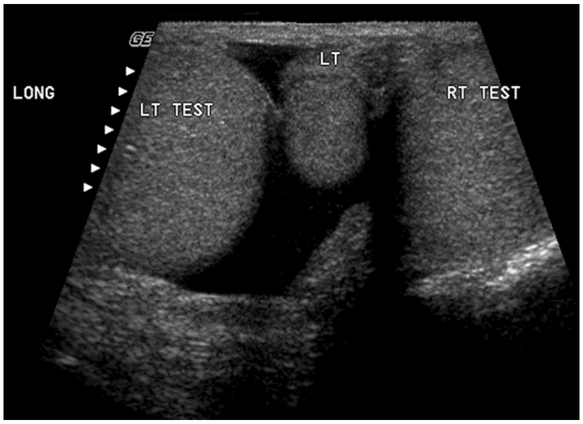

**FIGURE 1**. Scrotal ultrasound demonstrating supernumerary testis.

The patient's family history was significant for unspecified cancers and negative for all else, including genetic abnormalities. His medical history was significant for two separate episodes of renal stones, neither of which required intervention. He reported smoking one pack of cigarettes per day for the past 13 years, denied drinking alcohol or using recreational drugs, had no allergies, and took no medications regularly. Physical examination was largely unremarkable with the exception of a mass in his left hemiscrotum that felt distinct from the testis. His right testis measured 5.7 cm in long axis, his left testis measured 4.1 cm, and the mass measured 2.4 cm. There were no abnormalities noted with either the vas deferens or epididymis (both present bilaterally), or the penis. He had no other concerns aside from pain in the area of the left testicle. Tumor markers, including lactic acid dehydrogenase (LDH), human chorionic gonadotropin (hCG), and alpha-fetoprotein (AFP), were within normal limits. CT scan of the abdomen and pelvis revealed multiple tiny stones in the kidneys bilaterally; no stones were noted in the ureters or bladder. No signs of upper urinary tract obstruction were present. No seminal fluid analysis was done as part of the evaluation, as the patient was not married and had no desire for a conception at that time or in the near future.

After extensive counseling, the patient opted for surgical exploration and removal of the left scrotal mass, done under general anesthesia through an inguinal approach. The mass was found to be within the tunica vaginalis, ovoid in shape, and distinct from the left testis. Its outer surface had the appearance of tunica albuginea and it was not connected to an epididymis or vas deferens (Fig. 2). Frozen section examination was consistent with benign testicular tissue. The case was completed after hemostasis was confirmed and the left testicle returned to the scrotal compartment.

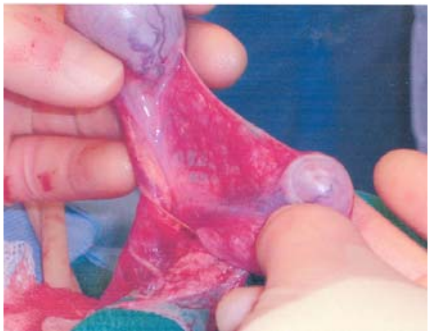

**FIGURE 2**. Intraoperative appearance of supernumerary testis.

Final pathology revealed a 1.7- × 1.5- × 0.9-cm tan ovoid mass, consistent with a supernumerary testis with Sertoli cell-only syndrome (germ cell aplasia) and Leydig cell hyperplasia. No plan for scheduled follow-up visits was made with regard to this anomaly since the mass was completely removed and found to be nonmalignant, and his remaining testes were left intact and he was otherwise healthy. The patient's postoperative course was uneventful. Coincidentally, he was followed for his nephrolithiasis and underwent a metabolic workup. He noted at that time that since surgery, he had had several episodes of fleeting, low-grade scrotal pain associated with heavy lifting that immediately subsided with cessation of activity.

## DISCUSSION

Several different systems of classification have been proposed for supernumerary testicles[5]. Functional classification includes: Type I, no vas deferens or epididymis communicates with the supernumerary testicle; Type II, the main testis and the supernumerary testis share both an epididymis and vas; and Type III, the main testis shares a vas with the supernumerary testis, which has its own epidydimis[5]. Classification has also been based on the location where the supernumerary testis is found — scrotum, peritoneum, retroperitoneum, or inguinal canal[6]. Using these two straightforward classifications, our case was Type I and scrotal.

Scrotal ultrasound is a very useful tool in the evaluation of scrotal masses, and while it may suggest the diagnosis of a supernumerary testicle, it will not obviate the need for surgery if associated with pain, torsion, or possible malignancy. A tissue diagnosis is required for definitive diagnosis[7].

Five cases of an association between supernumerary testis and malignancy have been reported[4]. This relatively small number has created a debate with regard to whether or not a supernumerary testicle should be removed. This decision is made more difficult as these testicles are usually asymptomatic. In our case, the impetus to remove the mass was greater as the patient was symptomatic and the nature of the lesion was unclear. Germ cell aplasia and associated scrotal pain are two unusual features of a nonfunctional supernumerary testis. As the question whether a supernumerary testis should be removed remains under debate, the decision should be made in a case-by-case fashion, taking into consideration the individual circumstances, such as the presence of local symptoms and concern for possible malignancy.
